# Smoking Initiation and Cessation among Youths in Vietnam: A Longitudinal Study Using the Chi Linh Demographic—Epidemiological Surveillance System (CHILILAB DESS)

**DOI:** 10.3934/publichealth.2017.1.1

**Published:** 2016-12-28

**Authors:** Duong Minh Duc, Le Thi Vui, Hoang Ngoc Son, Hoang Van Minh

**Affiliations:** 1 Hanoi School of Public Health, 138 Giang Vo Street, Ba Dinh District, Ha Noi, Vietnam; 2 International Maternal and Child Health (IMCH), Department of Women's and Children's Health, Uppsala University, SE-751 85 Uppsala, Sweden; 3 Department of General Surgery, Viet Duc University Hospital, 40 Trang Thi Street, Hoan Kiem District, Ha Noi, Vietnam

**Keywords:** smoking initiation, smoking cessation, youths, CHILILAB DESS

## Abstract

Study of smoking initiation and cessation is particularly important in adolescent population because smoking prevention and cessation at this time may prevent several health consequences later in life. There is a very limited knowledge about the determinants of smoking initiation and cessation among youths in Vietnam. This limits the development and implementation of appropriately targeted anti-smoking prevention interventions. This study applied pooled data from 3 rounds of a longitudinal survey in the Chi Linh Demographic—Epidemiological Surveillance System (CHILILAB DESS) in a northern province in Vietnam to analyse the determinants of smoking initiation and cessation among youths. The total of youths in the first round, second, and third rounds was 12,406, 10,211, and 7,654, respectively. The random-effects logit model controlling for both time-variant and time-invariant variables was conducted to explore the associated factors with new smokers and quitters. We found an increase trend of new smokers (7.0% to 9.6%) and quitters (27.5% to 31.4%) during 2009–2013. Smoking initiation and cessation are the result of multifactorial influences of demographic and health behaviours and status. Demographic background (older youths, male, unmarried youths, and youths having informal work) and health behaviours and status (youths who had smoking family members and/or smoking close friends, and had harmful drinking) were more likely to initiate smoking and more difficult to quit smoking. Among these variables, youths who had smoking close-friends had the highest likelihood of both initiating smoking and failed quitting. Our results could represent the similar health problems among youths in peri-urban areas in Vietnam. Further, our findings suggested that anti-smoking interventions should involve peer intervention, integrated with the reduction of other unhealthy behaviours such as alcohol consumption, and to focus on adolescents in their very early age (10–14 years old).

## 1. Introduction

During the twentieth century, smoking rose to epidemic proportions and has become a leading cause of premature death and disability [1]. Every year about six million deaths worldwide are attributed to tobacco use and exposure to second hand smoke [1]. Smoking is still rising globally, largely because of the emerging of targeted advertising toward youths and women in many low- and middle-income countries (LMICs) [1]. Further, smoking is not only a stand-alone disease but co-occurrence to many other health problems such as use of alcohol [2], and depression [3],[4] in LMICs. In response to this epidemic, the World Health Organization has called to develop scientifically-based research evidence, an important step to assist in tobacco control efforts [5].

Adolescence is considered as the key period for studying smoking initiation and cessation [4]. Most people start smoking before the age of 18. Aiming to effectively monitor the smoking rate among youths, the Global Youths Tobacco Survey has been developed and provided a lot of useful information for LMICs over the past decade [1]. For example, young men smoke more than young women both in overall consumption and in prevalence [6]. In many LMICs, at all levels of income, it is the poorest group who smokes the most, and bears most of the economic and disease burden of tobacco use [7],[8]. Moreover, the poorest group spend a significant more money on tobacco than on education and health care [8]. While there are some examples of cross-sectional studies exploring social determinants of smoking among youths, there is very little information about the smoking initiation and cessation among adolescents and youths in these settings.

Similar to other LMICs, smoking is one of the three leading factors for premature death and disability in Vietnam [9]. Smoking is the leading cause of preventable death among youths, estimated to cause more than 40,000 deaths a year, and this is predicted to rise [10]. Moreover, few studies offer evidences that explore the association of stages of smoking initiation, or patterns of cessation attempts among youths in Vietnam. Specifically, there are no current longitudinal studies, to the best of our knowledge, reporting the association of demographic and socioeconomic determinants of smoking initiation and cessation among adolescents and youths in Vietnam.

Society's treatment of smoking is complex and has radically changed over the years in Vietnam. A number of different measures have been issued and enforced in tobacco control programmes to reduce the prevalence of use in Vietnam [6],[11]. Tobacco advertising, for examples, is prohibited in all print and electronic media, excluding at the point-of-sale [12]. Following the WHO Framework Convention on Tobacco Control, Vietnamese government has required pictorial health warnings on all tobacco packages since 2013 [13]. Vietnam has also imposed a special tax on cigarettes, 70% of the retail price in 2016, in addition to the regular excise tax [13]. There have been, however, low proportions of quitters in Vietnam [6]. Understanding the factors that influence smoking initiation and cessation may provide valuable information for efforts to reduce smoking-related morbidity and mortality. Therefore, this study aimed to assess the association of demographic determinants and some health behaviours and status, such as alcohol drinking and depression, with smoking initiation and cessation among adolescents and youths in a peri-urban area in Vietnam during 2006–2013.

## 2. Methods

### 2.1. Study Setting

The study was conducted in the Chi Linh Demographic- Epidemiological Surveillance System (CHILILAB DESS) in Chi Linh, a peri-urban district in a northern province in Vietnam. This district covers an area of about 300 square kilometres including 12 communes and 8 towns with about 175,000 residents by 2015 [14]. The population density varies with a higher density in lowland communes. Approximately a fourth of the total population lives in urban areas.

The CHILILAB provides data on an urban and rural population, which is in view of the rapid economic and population growth in Vietnam. The socio-economic development and geographic characteristic as well as the health problems in this peri-urban district are considered to be representative of similar settings in Vietnam [15]. In addition to collecting quarterly basic health and demographic data, CHILILAB was designed to collect data on adolescent health. The adolescent health survey, which was conducted in 7 communes/towns in Chi Linh, to better understand the current situation in Vietnam with regard to youths' health, associated behaviours and the related antecedents that both predispose youths to risk and determinants that diminish health compromising behaviours.

### 2.2. Study Design & Population

This was a longitudinal cohort study among all youths aged 10–24 years old in 7 communes/towns in Chi Linh. Data was collected in three rounds during 7 years (2006–2013) at every 3-year interval from the same adolescents. Youths, who did not participate in three rounds, were either sick or did not stay in their house during the survey. The first, second, and third rounds of the survey were respectively conducted during July 2006 to January 2007, February to July 2009, and September to December 2013. The duration between the second and third round was longer than the duration between the first and second round due to a delay in survey organization. Virtually all youths had 7 years between the first and last survey. The three rounds included information from the household surveys, for example, sex, education level, and household economy using DESS questionnaires. The household surveys, which have been conducted biennially at both the household and individual levels since 2004, covered approximately 18,000 households in Chi Linh.

### 2.3. Data Collection

The current study used existing adolescent health survey data. The survey included three surveys, that is, health status and behaviour survey, risk and protective factors survey, and parent/family survey. The health status and behaviour survey was used for the analysis in this paper to obtain an accurate picture of the current health status of youths, experience with adverse health-related events, and risk-taking and health-seeking behaviours.

Data collection was done by trained female interviewers, who were also the data collectors of the CHILILAB DESS. They were local residents who had been selected by a thorough recruitment process. Most of these data collectors participated in all three rounds of survey as the turnover rate was as low as 10%. Several measures were conducted to ensure the quality of the data. A separate quality-control superiors re-surveyed 5% of the households and the main survey data was cross-checked with the re-survey findings to valid of the survey data. Any differences were re-checked and discussed to reach consensus.

### 2.4. Study Variables

#### 2.4.1. Outcome Variables

To assess *current smoking*, youths were asked how often, if ever, they had used cigarettes. The answers were then categorized into “never smoked” and “ever smoked in the last 30 days”. *Current smoking* was defined as those who reported to have used at least 1 cigarette in the past 30 days.

*New smoker* was defined as the transition from “never smoked” to *current smoking* at either follow-up.

*Quitter* was defined as the transition from *current smoking* to “not smoked cigarettes” at either follow-up in the past 30 days.

#### 2.4.2. Independent Variables

We selected and divided 11 potential independent variables relating to smoking initiation and cessation into: (1) demographic background: sex, age, marital status, occupation, household income, educational level; and (2) health behaviours and status: harmful drinking, depression status, and family member and peer influences (family members' smoking, close-friends' smoking, and family members' drinking). All these potential variables were evaluated in the present study.

Sex was composed of two categories: male and female. Age was categorised into three age groups of 10–14, 15–19, and 20–24 years as suggesting of the World Health Organization [5]. Marital status was dichotomised into married or single and the single group included those who were divorced. Household income was divided into two groups: poor and near poor (households that earn less than 1000 USD annually) and middle or higher income (households that earn more than 1000 USD annually). Current occupation was comprised of three groups: informal work (farmer, housework, and craft worker), student (students and vocational/work training) and others. Educational level was divided into two categories in the order of increasing level of education: high-school graduation or below and university degree or above. Setting was categorised as: rural and urban areas.

In term of health behaviours and status, similar to our previous publication, peer influences (family members' smoking, close-friends' smoking, family members' drinking) was categorized into “yes” or “no” by asking the youths about the cigarette smoking habits of their family members' smoking or drinking behaviour [2]. Harmful drinking, which was using the question, “Have you ever gotten into trouble because of drinking”, was also defined as those who reported that they had gotten into trouble because of drinking [2].

The depression status was assessed using a 16-item questionnaire. This depression scale was translated from the Centre for Epidemiological Studies—Depression Scale (CES-D) in the United States and adapted and tested for validity and reliability in the Chi Linh district context [16],[17]. The depression score was the sum of these items and then classified as either “yes” or “no” using a cut-off set at >40 and ≤40.

### 2.5. Data Analysis

The data analyses were carried out in two regression models using Stata version 13. The primary model was to explore the empirical relationship between independent variables and the outcome measures among youths aged 15–24 years old using the logistic regression model per round. The final model which used random-effects logit model at individual level for panel data was computed to control for both time-variant and time-invariant variables in all three rounds. Variables included in our analysis were dependent variable (new smoker and quitter), and independent variables (sex, age, marital status, occupation, household income, educational level, family members' smoking, close-friends' smoking, family members' drinking, harmful drinking and depression). *p*-value < 0.05 was considered as statistical significance.

### 2.6. Ethical Considerations

Ethical approval for this study was obtained from the Institutional Review Board of Ha Noi School of Public Health. All respondents were asked to participate in the survey by signing informed consent document. Further caregivers' agreement was also collected if the adolescents were under 18 years of age. Respondents could refuse to participate in or withdraw from the interview at any time. Respondents were given a small present for their participation.

## 3. Results

### 3.1. General Characteristics of the Study Respondents

The total sample size was about 20% lower after each round, ranging from a high of 12,406 in 2006 to a low of 7,654 in 2013. The sample divided equally in terms of sex and setting. The mean age of our youths by 2006 was 16.5 (SD = 3.9). There were changes overtime amongst sub-groups of other variables, including decreases in unmarried youths (from 91.4% to 72.8% during 2006–2013), decreases in being student (from 70.9% to 41.7% during 2006–2013), decreases in having high school or lower level (from 89.4% to 57.9% during 2006–2013), and increases in poor and near-poor households (27.8% in 2006 to 38.2% in 2013). In terms of peer and family members' smoking, about half of the youths had a smoking family member and a close-friend. About other health behaviours and status, a fourth of youths had harmful drinking by 2013. Notably, a fourth of the youths had some depression symptoms. For further details regarding Demographic Background, see Table 1.

**Table 1.** Demographic Background and Health behaviours and status among Youths: Three rounds of survey 2006–2013 in CHILILAB DESS.

Variables (N, %)		2006		2009		2013	
		n	%	n	%	n	%
	Overall	12,406		10,211		7,654	
**Demographic background**							
**1**	**Sex**						
	Male	6,091	49.1	4,996	48.9	3,678	48.1
	Female	6,315	50.9	5,215	51.1	3,976	52.0
**2**	**Age**						
	10–14 years old	4,237	34.2	4,024	39.4	3,067	40.1
	15–19 years old	5,047	40.7	3,884	38.0	2,776	36.3
	20–24 years old	3,122	25.2	2,303	22.6	1,811	23.7
**3**	**Marital status**						
	Unmarried	11,339	91.4	8,420	84.4	5,509	72.8
	Married	1,067	8.6	1,553	15.6	2,059	27.2
**4**	**Occupation**						
	Informal work	1,859	15.2	1,413	13.8	1,177	15.4
	Pupils/Students	8,678	70.9	6,736	66.0	3,193	41.7
	Others	1,708	14.0	2,061	20.2	3,284	42.9
**5**	**Household income**						
	Poor and near poor	3,393	27.8	2,815	35.8	1,633	38.2
	Middle or higher income	8,794	72.2	5,040	64.2	2,643	61.8
**6**	**Education level**						
	High school or lower level	10,876	89.4	8,510	83.4	4,431	57.9
	College/university degree or higher	1,284	10.6	1,700	16.7	3,223	42.1
**7**	**Setting**						
	Rural	6,397	51.9	5,291	51.8	4,018	53.9
	Urban	5,925	48.1	4,920	48.2	3,433	46.1
**Health behaviors and status**							
**8**	**Family members' smoking**						
	No	5,395	43.5	4,332	42.4	3,737	48.8
	Yes	7,011	56.5	5,875	57.6	3,916	51.2
**9**	**Close-friends' smoking**						
	No	6,494	55.7	4,719	47.1	4,064	53.1
	Yes	5,156	44.3	5,303	52.9	3,588	46.9
**10**	**Family members' drinking**						
	No	7,841	63.2	6,702	65.7	6,173	81.2
	Yes	4,565	36.8	3,503	34.3	1,434	18.9
**11**	**Harmful drinking**						
	No	8,483	75.3	7,227	72.6	5,519	73.2
	Yes	2,783	24.7	2,735	27.5	2,019	26.8
**12**	**Depression**						
	No	10,740	86.57	7,436	72.82	5,873	76.73
	Yes	1,666	13.43	2,775	27.18	1,781	23.27

### 3.2. Prevalence of Current Smokers, new Smokers and Quit Smoking

The prevalence of *current smokers, new smokers,* and *quitters* among youths in CHILILAB increased overtime. The prevalence of *current smokers* increased slightly from 11.2% in round 1 (2006) to 17.6% in round 3 (2013). Across all demographic background but not education levels, there was a significant increase in rate of *new smokers* among youths (Table 2). Smoking rate among females, for example, had doubled among female during the study period. Smoking prevalence fell among youths who attended college/university degree. Two sub-groups of youths did not experience increase in prevalence rate, those aged 15 to 19 and those who had informal work. Similar to rate of *new smokers*, quit rate amongst youths increased from 27.5% in round 2 (2009) to 31.4% in round 3 (2013). The trend rate of *quitters*, however, was mixed among sub-groups of demographic variables. Three sub-groups with a significant increase in quitters were youths aged 15–19 years old (23.9% to 32.6%), and pupils/students (40.3% to 57.6%). Only one sub-group, youths aged 10–14 years old, had a significant decrease rate of *quitters*. The remaining sub-groups had quite small changes of *quitters* overtime.

**Table 2.** Distribution of Smoking Initiation and Smoking Cessation among Youths by Demographic Background and Health behaviours and status: Three Rounds of Survey 2006–2013 in CHILILAB DESS.

Variable (N, %)			Current Smokers			New Smokers		Quitters	
**Years**			**2006**	**2009**	**2013**	**2009**	**2013**	**2009**	**2013**
**Total**			**1,350**	**1,304**	**1,279**	**553**	**559**	**222**	**276**
			**11.2**	**13.6**	**17.6**	**7.0**	**9.6**	**27.5**	**31.4**
**Demographic background**									
**1**	**Sex**								
	Male		1,274	1,285	1,228	541	534	180	265
			21.5	28	36.3	15.7	24.6	23.7	30.6
	Female		76	19	51	12	25	42	11
			1.3	0.4	1.3	0.3	0.7	93.3	84.6
**2**	**Age**								
	10–14 years old		93	149	350	126	252	60	56
			2.2	3.9	12	3.6	9.6	80.0	49.6
	15–19 years old		549	609	507	307	207	78	130
			11.2	16.9	19.3	10.3	10.2	23.9	32.6
	20–24 years old		708	546	422	120	100	84	90
			23.8	24.9	24.6	8.7	8.8	20.7	24.5
**3**	**Marital status**								
	Unmarried		1,219	1,051	856	505	444	183	199
			11.1	13.3	16.4	7.5	10.4	29.7	37.3
	Married		131	242	417	41	109	37	73
			12.7	16.2	21.2	4.1	7.6	20.0	21.4
**4**	**Occupation**								
	Informal work		444	414	357	132	118	61	60
			25	30.7	32	15.1	15.6	21.0	22.3
	Pupils/Students		537	469	254	290	183	96	57
			6.4	7.5	8.4	5.1	6.7	40.3	57.6
	Others		342	421	668	131	258	65	159
			20.9	21.5	21.4	9.7	11.1	23.4	31.1
**5**	**Household income**								
	Poor and near poor		417	365	349	150	149	66	52
			12.8	13.7	22.3	6.9	12.1	29.1	23.5
	Middle or higher income		913	553	394	241	172	105	94
			10.7	11.6	15.8	6.1	8.6	31.1	35.1
**6**	**Education level**								
	High school or lower level		1,106	1,017	883	441	383	192	180
			10.5	12.7	21	6.6	11.6	30.3	29.7
	College/university degree or higher		226	287	396	112	176	30	96
			18.4	18.3	13	9.2	7.1	17.3	35.3
**7**	**Setting**								
	Rural		745	710	780	308	366	138	145
			12.1	14.1	20.2	7.4	11.6	29.7	28.7
	Urban		599	594	467	245	193	84	131
			10.4	13	14.5	6.6	7.3	24.6	35.1
**Health behaviors and status**									
**8**	**Family members' smoking**								
	No		469	480	512	210	229	112	153
			9.1	11.7	14.4	6.2	8.0	35.8	39.6
	Yes		881	823	767	342	330	110	123
			12.8	14.9	20.8	7.6	11.3	22.3	25.0
**9**	**Close-friends' smoking**								
	No		207	157	270	76	127	80	142
			3.2	3.5	6.9	1.9	3.8	58.4	54.6
	Yes		1,082	1,126	1,008	469	432	135	134
			21.5	22.9	30.0	12.4	17.5	20.7	21.7
**10**	**Family members' drinking**								
	No		469	480	512	329	416	158	233
			9.1	11.7	14.4	6.3	8.8	30.4	33.5
	Yes		881	823	767	223	137	64	42
			12.8	15.0	20.8	8.2	13.0	22.4	23.3
**11**	**Harmful drinking**								
	No		310	373	508	178	248	124	182
			3.7	5.4	9.6	3.0	5.5	46.4	45.4
	Yes		818	812	703	319	288	93	88
			30.2	33.0	38.0	18.3	23.1	19.3	20.1
**12**	**Depression**								
	No		1145	926	998	383	440	160	227
			11.0	13.2	17.8	6.6	9.9	27.5	32.7
	Yes		205	378	281	170	119	62	49
			12.6	14.5	16.9	8.0	8.9	27.6	26.5

### 3.3. Factors Associated with Smoking Initiation and Cessation

The final random-effects binary logistic model confirmed the determinants of *new smokers* and *quitters* among youths (Table 3). Among 6 demographic variables, only two demographic variables (sex and occupation) showed a differential effect smoking initiation, whereas being female was the strongest protective factor against initiating smoking (OR = 0.02, CI: 0.01–0.04). In term of smoking cessation, four variables showing a significant association (sex, age, marital status, and occupation). The odds of successfully quitting smoking increased among female youths (OR = 7.79, CI: 3.16–19.20) and youths who were students (OR = 1.95, CI: 1.18–3.22) or youths who had other work (OR = 1.56, CI: 1.06–2.31). Older youths (OR = 0.34) and married youths (OR = 0.55, CI: 0.36–0.83) were associated with a lower likelihood of smoking cessation.

In terms of health behaviours and status behaviours, three variables family members' smoking status, close friends' smoking status, and harmful drinking were associated with both smoking initiation and successfully smoking cessation. Among these variable, youths who had smoking close friends had the greatest likelihood of initiating smoking (OR = 3.43, CI: 2.63–4.48) and failed quitting (OR = 0.33, CI: 0.22–0.47). Harmful drinking was also a potent determinant of smoking status as high odds of initiating smoking (OR = 2.89, CI: 2.26–3.68) and failed quitting (OR = 0.43, CI: 0.30–0.59).

**Table 3.** Results of Random-Effects Logit Models for Smoking Initiation and Smoking Cessation by Demographic Background and Health behaviours and status: Three Rounds of Survey 2006–2013 in CHILILAB DESS.

Variables		New Smokers			Quitters		
**Demographic background**		**OR**	**Lower CI**	**Upper CI**	**OR**	**Lower CI**	**Upper CI**
**1**	**Sex**						
	Male	Ref	-	-	Ref	-	-
	Female	**0.02 ^c^**	**0.01**	**0.04**	**7.79 ^c^**	**3.16**	**19.20**
**2**	**Age**						
	10–14 years old	Ref	-	-	Ref	-	-
	15–19 years old	1.15	0.89	1.47	**0.34 ^c^**	**0.20**	**0.57**
	20–24 years old	0.83	0.55	1.24	**0.34 ^c^**	**0.19**	**0.61**
**3**	**Marital status**						
	Unmarried	Ref	-	-	Ref	-	-
	Married	1.28	0.85	1.93	**0.55 ^b^**	**0.36**	**0.83**
**4**	**Occupation**						
	Informal work	Ref	-	-	Ref	-	-
	Pupils/Students	**0.20 ^c^**	**0.14**	**0.28**	**1.95 ^a^**	**1.18**	**3.22**
	Others	**0.65 ^b^**	**0.47**	**0.88**	**1.56 ^a^**	**1.06**	**2.31**
**5**	**Household income**						
	Poor and near poor	Ref	-	-	Ref	-	-
	Middle or higher income	0.88	0.71	1.09	1.04	0.73	1.49
**6**	**Education level**						
	High school or lower level	Ref	-	-	Ref	-	-
	College/university degree or higher	1.01	0.75	1.36	0.84	0.55	1.28
**Health behaviors and status**							
**7**	**Family members' smoking**						
	No	Ref	-	-	Ref	-	-
	Yes	**1.26 ^a^**	**1.01**	**1.56**	**0.49 ^c^**	**0.35**	**0.70**
**8**	**Close-friends' smoking**						
	No	Ref	-	-	Ref	-	-
	Yes	**3.43 ^c^**	**2.63**	**4.48**	**0.33 ^c^**	**0.22**	**0.47**
**9**	**Family members' drinking**						
	No	Ref	-	-	Ref	-	-
	Yes	1.05	0.84	1.32	0.89	0.59	1.32
**10**	**Harmful drinking**						
	No	Ref	-	-	Ref	-	-
	Yes	**2.89 ^c^**	**2.26**	**3.68**	**0.43^c^**	**0.30**	**0.59**
**11**	**Depression**						
	No	Ref	-	-	Ref	-	-
	Yes	0.91	0.72	1.16	0.89	0.60	1.32

**^a^**: represents statistically significant at *p*-value <0.05; **^b^**: represents statistically significant at *p*-value <0.01; **^c^**: represents statistically significant at *p*-value < 0.001. **Abbreviations**: OR: Odd ratio; Ref: Reference group.

## 4. Discussion

This study used pooled data from 3 rounds of a longitudinal survey in the CHILILAB DESS in a northern province in Vietnam to analyse the determinants of smoking initiation and cessation among youths in Vietnam. This study, to our best knowledge, is the first longitudinal analysis with regards to smoking initiation and cessation among youths aged 10–24 years in Vietnam. We found increase trend of smoking initiation (7.0% to 9.6%) and smoking cessation (27.5% to 31.4%) overtime among a representative sample of youths for peri-urban areas in Vietnam. Smoking initiation and cessation was the result of multifactorial influences of demographic and health behaviours and status. Two demographic variables (sex and occupation) exerted influences on both smoking initiation and smoking cessation while age and marital status were strong determinants of failed quitting. Furthermore, we found family members' smoking status, close friends' smoking status, and harmful drinking were associated with higher likelihoods of both smoking initiation and smoking cessation.

### 4.1. Determinants of Smoking Initiation and Cessation amongst Youths in Vietnam

We found an inverse association of youths with older age and married youths to smoking cessation among youths. Consistent with this finding, previous studies reports that smokers initiated their smoking habit before the age of 19 [18],[19]. Smokers who have longer lifetime tobacco use appeared to have lower likelihood of changing their smoking status [20]. Furthermore, other studies also stress a substantial increase in initiating smoking during the transition from adolescence to young adulthood [18],[19]. The high smoking rate and low quit rate amongst older youths (15–19 years old and 20–24 years old) were worry some. These suggested that tobacco control initiatives in Vietnam should target at youths in their very early ages (10–14 years old) and unmarried youths.

Sex and occupation were also potent determinants of both smoking initiation and smoking cessation. Smoking has been repeatedly highlighted as the most pressing health problems in Vietnam but for males only [6],[21]. Our analysis highlighted concerns regarding female smoking, given that smoking prevalence has doubled among females during our study period. The increase trend of smoking among females has been also raised in previous reports in South-east Asian countries [19],[22]. In terms of occupation, smoking initiation was significantly lower among youths having informal work (15.6%) than students (6.7%) and youths having other work (11.1%). Moreover, the two latter groups (students and other work) were two times more likely to quit smoking. Similarly, other studies present that unemployed youths have significantly higher levels of smoking than students [23],[24].

Peer and parental smoking increase the risk of smoking among youths [25]–[27]. Although both peer influence and parental influence played an important role in determining the adolescents' smoking, close-friends' smoking status was the most important predictor to smoking initiation and cessation in our study. These findings are consistent with previous studies reporting that the impacts of peer smoking increase over lifetime to youths in Western culture [25]. Young non-smokers who have smoking friends were more likely to initiate smoking and difficulty quitting smoking than youths without any smoking friends [25],[27],[28]. These findings are different from a previous study among students in high school in Taiwan stating that parental influence, as a whole, is more important than peer influence [29].This implied a change in the role of parents regarding to smoking initiation and cessation of youths. One reason could be that parents have low levels of communication with their adolescents [30]. Moreover, our study also highlighted the co-occurrence of smoking and alcohol drinking and supported the negative association between alcohol drinking and successful smoking cessation. This finding is consistent with other studies showing that smokers are much more likely to be alcohol drinkers and that alcohol drinker is less likely to quit smoking [24],[31],[32]. This suggested that anti-smoking interventions should be integrated with anti- drinking interventions.

### 4.2. Suggestions to Establish Smoking Prevention and Cessation Program amongst Youths in Vietnam

Socioeconomic disadvantages, regarding to educational attainment and economic status, have been reported to be potential predictors of smoking behaviours amongst youths in previous studies [7],[20]. Lower educational attainment, for example, is associated with more likelihood of smoking initiation and less likelihood of quitting smoking [20],[33]. This is consistent with our results suggesting that background variables, which were, age, sex, education, and marital status, were likely to influence smoking initiation and cessation amongst youths in Vietnam. We, however, did not find a significant association between household income and smoking initiation and cessation. This could be partly explained because of the easy accessibility and low price which make cigarettes becoming more affordable overtime in Vietnam [34],[35]. Taxes on cigarettes might need to be further increased in Vietnam.

Combined all our findings related to determinants of smoking initiation and cessation, our study implied that strategic interventions should consider the individual factors of sex, age, marital status and occupation, health risk behaviour (alcohol consumption), and peer and family members' tobacco smoking and alcohol drinking. This agrees with other reports showing that anti-smoking interventions should not only focus in school-based approach but also include community-based interventions covering other harmful behaviours and targeting out-of-school adolescents [18],[36]. Further, prevention of smoking initiation in early adolescents should be a priority considering the lower likelihood of smoking cessation among older youths and extremely negative effects of smoking on their health later in life.

### 4.3. Methodological Considerations

The advantages of our study were the use of a large sample, representative institutional sampling frame, and retrospective cohort. Thus, the determinants of smoking initiation and cessation among youths in Chi Linh could represent all youths in similar peri-urban settings in Vietnam. Our findings, however, may not be generalized for youths living in extremely hard-to-reach areas and ethnic minority youths due to a small proportion of these groups in our survey (<0.5%). Besides, selection bias could occur in our study as a result of loss to follow-up. We lost about 20–25% of respondents in each following round due to sickness, relocation, or loss of interest in our study. The follow-up rate in our study was 61%, suggesting an acceptable follow-up rate [37].

## 5. Conclusions

Our findings have shown an increasing trend in smoking initiation and smoking cessation overtime among a cohort sample of youths in a northern province in Vietnam. The problems of smoking initiation and cessation among youths in our study could represent all youths in peri-urban in Vietnam. Demographic background (older youths, male, unmarried youths, and youths having informal work) and health behaviours and status (youths who had smoking family members and/or smoking close friends, and had harmful drinking) were more likely to initiate smoking and more difficult to quit smoking. Among these variables, close-friends' smoking status was the most important variable predicting smoking behaviour amongst youths. Our findings suggested that anti-smoking interventions should involve peer intervention, integrate with the reduction of other unhealthy behaviours such as alcohol drinking, and focus on adolescents in their very early age (10–14 years old).
